# Fine-needle aspiration for diagnosis of tuberculous lymphadenitis in children in Bangui, Central African Republic

**DOI:** 10.1186/1471-2431-12-191

**Published:** 2012-12-13

**Authors:** Minime-Lingoupou Fanny, Narcisse Beyam, Jean Chrusostome Gody, G Zandanga, F Yango, Alexandre Manirakiza, Leen Rigouts, Catherine Pierre-Audigier, Brigitte Gicquel, Gustave Bobossi

**Affiliations:** 1 Laboratoire des Mycobactéries, Institut Pasteur de Bangui, Bangui, Central African Republic (CAR; 2 Hôpital pédiatrique de Bangui, Bangui, CAR; 3 Mycobacteriology Unit, Institute of Tropical Medicine, and Department of Pharmaceutical, Veterinary and Biomedical Sciences, University of Antwerp, Antwerp, Belgium; 4 Unité de Génétique Mycobactérienne, Institut Pasteur, Paris, France

**Keywords:** Tuberculosis, Lymphadenitis, Fine needle aspiration, Diagnosis

## Abstract

**Background:**

Tuberculosis (TB) is a major cause of childhood morbidity and mortality in developing countries. One of the main difficulties is obtaining adequate specimens for bacteriological confirmation of the disease in children.

The aim of this study is to evaluate the adequacy of fine-needle aspiration (FNA) for the diagnosis of TB.

**Methods:**

In a prospective study conducted at the paediatric hospital in Bangui in 2007–2009, we used fine-needle aspiration to obtain samples for diagnosis of TB from 131 children aged 0–17 years with persistent lymphadenitis.

**Results:**

Fine-needle aspiration provided samples that could be used for bacteriological confirmation of TB. Ziehl-Neelsen staining for acid-fast bacilli was positive in 42.7% of samples, and culture identified TB in 67.2% of cases. Of 75 samples that were stain-negative, 49 (65.3%) were culture-positive, while 12 stain-positive samples remained culture-negative. Ten of the 12 stain-positive, culture-negative samples were from patients who had received previous antimicrobial therapy. With regard to phenotypic drug susceptibility, 81/88 strains (91.1%) were fully susceptible to isoniazid, rifampicin, ethambutol and streptomycin, six (6.8%) were resistant to one drug, and one multidrug-resistant strain was found.

**Conclusions:**

Fine-needle aspiration is simple, cost-effective and non-invasive and can be performed by trained staff. Combined with rapid molecular diagnostic tests, fine-needle aspirates could improve the diagnosis of TB and provide valuable information for appropriate treatment and drug resistance.

## Background

Tuberculosis (TB) is a major cause of childhood morbidity and mortality in developing countries ( [1,2]. Accurate figures on the prevalence of paediatric TB are not available because the health information systems in endemic countries are inadequate and limited attention is paid to children, who contribute little to TB transmission in affected communities. The World Health Organization (WHO) estimates of TB incidence are based on sputum smear-positive cases, but more than 80% of children with TB are sputum smear-negative, and extrapulmonary TB is common in these patients. According to WHO, the evaluation of new techniques to improve the diagnosis and management of paediatric TB is an urgent research priority [3].

Like most sub-Saharan countries, the Central African Republic (CAR) pays a heavy price in terms of TB morbidity and mortality, with an annual incidence of all forms of TB estimated in 2009 at 345 per 100 000 [4] and a death rate of 113 per 100 000 inhabitants [5]. The situation is aggravated by co-infection with HIV, also in children: in a study conducted in 2005 [6], 25.7% of children aged 18 months to 15 years being treated for TB in the paediatric clinic in Bangui were co-infected with HIV. In the same study, fine-needle aspirates showed that lymphadenitis was common among cases of extrapulmonary TB.

Tuberculous lymphadenopathy is a common cause of peripheral adenopathy among children [7], and lymphadenopathy is a common clinical symptom of extrapulmonary TB in the pediatric age group, responsible for up to 50% of all extrathoracic TB [8,9]. In endemic areas, TB is the commonest cause (22–48%) of persistent cervical lymphadenopathy [10].

Fine-needle aspiration is a simple, safe outpatient procedure that can be performed by nurses in resource-limited settings. It provides material for direct microscopy, culture and susceptibility testing, thus improving bacteriological diagnosis [11]. It nevertheless remains a greatly underused means of collecting specimens and has not been used systematically in CAR.

The objective of this study was to evaluate the adequacy of fine-needle aspirates from enlarged lymph nodes for the diagnosis of clinically suspected childhood TB.

## Methods

### Study population

A prospective study was conducted between 2007 and 2009 at Unit D of the Paediatric Hospital of Bangui. The main inclusion criterion was the presence of adenitis. Thus, all children aged 0–17 years who had had lymphadenitis of unknown etiology for over 1 month were included. Lymphadenitis was defined as a painless firm or soft swelling in a group of superficial lymph nodes [12]. Parents or guardians gave their written informed consent for study participation. Ethical approval was obtained from the ethics committee of the CAR.

Tuberculin skin testing was performed in several children; however, because some children had been vaccinated and the HIV serological status of all the children was not known, we could not take into account the partial data obtained from skin testing.

### Sampling

In general, parents or guardians held the child during the procedure. Fine-needle aspiration was performed by a paediatric doctor using an 18-G needle (1.2 x 40 mm), who fixed the enlarged lymph node and inserted the needle in various directions. A different needle was used for each affected node, with a maximum of two per patient. The needle and syringe were immediately sent to the Institut Pasteur of Bangui for Ziehl-Neelsen staining for detection of acid-fast bacilli by optical microscopy, culture on Löwenstein-Jensen medium to identify *Mycobacterium tuberculosis* and testing for drug susceptibility.

### Identification and drug-susceptibility testing

*M. tuberculosis* was identified on the basis of biochemical and morphological criteria: growth rate, colony morphology, resistance to 2 mg/ml hydrazide acid thiophene-2 carboxylic acid, sensitivity to 0.5 mg/ml *para*-aminosalicylate and 10 mg/ml thiosemicarbazone, niacin production, nitrate reduction and catalase production. Susceptibility to anti-TB drugs was determined by the indirect proportion method [13] from the critical concentrations of isoniazid (0.2 μg/ml), rifampicin (40 μg/ml), ethambutol (2 μg/ml) and streptomycin (4 μg/ml).

## Results

Of 131 children who presented with suspected tuberculous lymphadenitis, nearly half (45.8%) were aged 0–5 years, and 69 (53%) had known contact with active TB within the family. All the children had general symptoms including fever (92%), asthenia (61%) and weight loss (88%). Most of the children (76%) presented with enlarged posterior cervical lymph nodes; the other locations were axillary (50%), inguinal (46%) and submandibular (32%) (Table 1); 97 (74%) children showed swelling of multiple nodes. HIV serology was performed in 103 children, and 36 (35%) were found to be seropositive, 63 (61%) seronegative and 4 (4%) of unclear HIV status.

**Table 1:** Demographics and sample charactereristics of children in whom FNA were performed (n=131)

**Demographics**	**n (%)**
Age, years	
0-5	60 (45.8)
6-10	36 (27.5)
11-14	24 (18.3)
15-17	11 (8.4)
Sex	
Male	68 (52)
Female	63 (48)
HIV status	
Positive	36 (35%)
Negative	63 (61%)
Unknown	4 ( 4%)
Site of lymph nodes	
Cervical	99/131(76%)
Axillary	66/131 (50%)
Other	102/131 (78%)
Contact with familiy TB	66/131 (53%)

Fine-needle aspiration was performed on one or two enlarged lymph nodes from each child. Ziehl-Neelsen staining was positive for 56/131 (42.7%) cases (Table 2); of these, 49.5% patients presented multiple enlarged nodes and 23.5% a single node. Of the Löwenstein-Jensen cultures, 88 (67.2%) were positive, 41 were negative and two were discarded because of contamination. Of 75 samples that were negative for acid-fast bacilli, 49 (65.3%) were positive by culture, while 12 stain-positive samples remained culture-negative.

**Table 2:** Outcome of bacteriological diagnosis for FNA samples taken from 131 children with tuberculous lymphadenitis

**Direct smear microscopy**	**Culture on Löwenstein-Jensen medium**		**Total**
	**Positive**	**Negative**	
Positive	39	17	56
Negative	49	24	73
Total	88	41	129*

*2 strains were contaminated.

Previous treatment of the patients was determined from clinical data. Ten of the 12 stain-positive samples with a negative Löwenstein-Jensen culture were from patients who had received antimicrobial therapy before fine-needle aspiration. All 88 positive cultures were identified as belonging to the *M. tuberculosis* complex. No nontuberculous mycobacteria were identified. With regard to phenotypic drug susceptibility, 81/88 strains (91.1%) were fully susceptible to isoniazid, rifampicin, ethambutol and streptomycin, six (6.8%) were resistant to one drug, and one multidrug-resistant strain was found (Table 3). The child (11 years) with a phenotypic multidrug-resistant strain had a history of prior TB treatment.

**Table 3:** Drug-susceptibility results for 88 *M. tuberculosis*-complex isolated from 131 children with tuberculous lymphadenitis

**Resistance profile**	**N (%)**
Susceptible to all drugs	81 (91.1)
Resistant to INH only	3 (3.4)
Resistant to SM only	3 (3.4)
MDR	1 (1.1)
Total	88

More bacteriologically confirmed TB cases were found among HIV-seronegative (45/63, 71%) than HIV-seropositive children (Table 4).

**Table 4:** Culture results in relation to HIV status

**HIV-positive n/N (%)**	**HIV-negative n/N (%)**	**P value**	**HIV-positive**
Culture-positive	18/63 (29%)	45/63 (71%)	0.0055
Culture-negative	18/36 (50%)	18/36 (50%)	

## Discussion

This study confirms the usefulness of fine-needle aspiration for investigating patients with suspected tuberculous lymphadenitis before starting anti-TB therapy. Microscopy showed the presence of potential TB bacilli in 42.7% of the aspirates, and culture identified TB in 67.2% of cases. The percentage of Ziehl-Neelsen-positive strains was much higher than the 18% observed by Knox [14] but close to those reported recently (45% [15] and 48.2–51.8% [16]) with light-emitting diode microscopy in studies of mycobacterial lymphadenitis in fine-needle aspirates from children. The conventional Ziehl-Neelsen method on smears is widely used and plays a key role in TB diagnosis, but it has a poor sensitivity in aspirates because of the small number of mycobacterial cells. Löwenstein-Jensen culture showed that 88 (67.2%) samples were positive, confirming the greater sensitivity of culture than Ziehl-Neelsen staining, and in agreement with recent reports of culture sensitivity of 65.8% [15], 69% [16] and 86.4% [14].

Our finding that 83.3% of smear-positive culture-negative specimens were from patients who had received anti-TB treatment suggests that positive acid-fast bacilli results might correspond to dead bacilli from previously treated patients. This confirms the need to perform bacteriological diagnosis before treatment. Mycobacterial culture is the gold standard for detecting tubercle bacilli, although it is time-consuming and requires specialized technology and procedures in a biosafety facility.

The study confirmed that the overall drug resistance rate is close to that reported in CAR between 1998 and 2000 [6] and is lower than the rate published by Koeck et al. [17] in Djibouti. Resistance to isoniazid and streptomycin was most frequent in children with undiagnosed TB, which confirms our previous findings in adults [18]. In CAR, these drugs are improperly used for the treatment of other bacterial infections, which can lead to resistance.

In general, biopsy is the preferred method for obtaining a sample for testing. In most of sub-Saharan Africa, however, due to a lack of facilities and manpower, it is feasible for only very few patients. Lymph node aspirates are simple to obtain, and the procedure is cheap and safe with limited risks, as it is less invasive and requires minimal instrumentation. This technique is especially suitable for peripheral lymph nodes and can be performed by trained nurses in small hospitals and clinics.

Recent advances in molecular diagnosis will affect future TB diagnostic approaches. Detection of mycobacterial DNA and rifampicin resistance with nucleic acid-based methods can be helpful in the diagnosis of tuberculous lymphadenatis. Combining fine-needle aspiration, which can be performed on an outpatient basis in a primary health care setting, with a rapid, sensitive diagnostic technique such as those based on nucleic acids may contribute substantially to the effective management of mycobacterial infection in children [19,20]. The implications of rapid, accurate diagnosis include access to appropriate, adequate therapy and less costly further investigations. The fully automated real-time polymerase chain reaction-based GeneXpert MTB/RIF test allows rapid, highly sensitive detection of *M. tuberculosis* complex DNA and of mutation-mediating rifampicin resistance [21], and WHO recently endorsed this new diagnostic test [22]. When used on lymph node tissue, the reported sensitivity for bacterial diagnosis was 86% [19] to 96% [20]. This new test and other, more effective, less costly tests being developed will strengthen rapid diagnosis from fine-needle aspirates. Rational use of fine-needle aspiration followed by immediate empiric therapy is the optimal approach. Although the GeneXpert MTB/RIF test may be useful for rapid detection of TB in lymph node aspirates and can indicate multidrug-resistant TB in cases of rifampicin resistance, it cannot, however, replace other tests for precise identification of other antibiotic resistance or confirm rifampicin resistance.

## Conclusions

The use of fine-needle aspiration, combined with new, rapid molecular diagnostic tests, could improve the diagnosis of tuberculous lymphadenopathy and provide valuable information for appropriate treatment. In this study, we were unable to assess complications such as haemorrhage, secondary infection or the development of sinuses, and these aspects should be considered in further studies to ensure that the procedure is safe and to define the rate of complications.

## Competing interests

The authors declare that they have no competing interests for this work.

## Authors’ contributions

FM-L, NB and AM wrote the manuscript. LR, CP-A and BG revised it critically for intellectual content, and JCG and GB gave final approval of the version to be published. All authors read and approved the final manuscript.

## Pre-publication history

The pre-publication history for this paper can be accessed here:

http://www.biomedcentral.com/1471-2431/12/191/prepub
